# Phase separation of Snail1 modulates transcription and cancer progression

**DOI:** 10.1016/j.gendis.2026.102147

**Published:** 2026-03-17

**Authors:** Yi-Ping Huang, Peng-Wei Zhang, Ke He, Zhong-Bao Shao, Wei-Jing Liu, You-Qing Zhu, Shi-Jie Zeng, Zhi Shi

**Affiliations:** Cancer Minimally Invasive Therapies Centre, Guangdong Second Provincial General Hospital, Department of Cell Biology & Institute of Biomedicine, Guangdong Provincial Biotechnology & Engineering Technology Research Center, Guangdong Provincial Key Laboratory of Bioengineering Medicine, Guangdong Basic Research Center of Excellence for Natural Bioactive Molecules and Discovery of Innovative Drugs, Genomic Medicine Engineering Research Center of Ministry of Education, MOE Key Laboratory of Tumor Molecular Biology, National Engineering Research Center of Genetic Medicine, State Key Laboratory of Bioactive Molecules and Druggability Assessment, College of Life Science and Technology, Jinan University, Guangzhou, Guangdong 510632, China

Liquid–liquid phase separation (LLPS, commonly referred to as phase separation) is a dynamic and reversible process that spontaneously leads to the separation of a homogeneous solution of biomolecules, including proteins or nucleic acids, into at least two coexisting phases: a dense phase enriched with the biomolecules, forming large droplets via weak or moderate affinity and multivalent interactions, and a surrounding dilute phase depleted of the biomolecules. Various factors influence phase separation, including pH, pressure, temperature, ion concentration, and environmental stress. Cellular components can form membrane-less biomolecular condensates through phase separation, thereby partitioning the cell into smaller compartments that increase molecular collision frequency and accelerate reaction rates.^1^ Phase separation gives rise to distinct states of biomolecular condensates, such as liquids, gels, and solids. Biomolecular condensates are critical intracellular regulators of numerous biological processes, including chromatin organization, DNA damage response and repair, transcriptional regulation, protein homeostasis, signal transduction, and material metabolism. Phase separation of proteins is facilitated by two structural features: multivalent folded domains and intrinsically disordered regions (IDRs) that contain multiple interacting motifs.^2^ Cancer progression is regulated by the formation of phase separation-mediated biomolecular condensates, which operate by affecting the function and expression of oncogenes and tumor suppressor genes, modulating proliferation, migration, invasion, and metastasis, and altering the tumor microenvironment and drug sensitivity.^3^^,^^4^ Snail1 is a member of the Snail family of zinc-finger transcription factors. The human Snail1 protein is a C2H2 zinc-finger protein consisting of 264 amino acids. Snail1 transcriptionally controls the expression of E-cadherin and other genes to regulate epithelial–mesenchymal transition, a process by which epithelial cells acquire a mesenchymal phenotype due to loss of their apical polarity, leading to an increased ability of migration and invasion. The expression of Snail1 is increased in a variety of cancer cells and tissues, and is a valuable indicator of poor prognosis in multiple cancers. The significance of Snail1 as a prognostic biomarker and its roles in cancer demonstrate that Snail1 is an attractive target for cancer therapy.^5^ Although Snail1 has been extensively studied, it remains unclear whether Snail1 is capable of undergoing phase separation.

To test whether Snail1 undergoes phase separation, we expressed human Snail1 protein fused with the fluorescent protein Venus in human embryonic kidney cells HEK293T, human hepatocellular carcinoma cells MHCC97H, and human gastric cancer cells MGC803. As shown in Fig. S1A–S1D, Venus-Snail1, but not Venus alone, could form condensates in these cells. Treatment with sorbitol or PEG8000 increased the area and fusion of Venus-Snail1 condensates (Fig. S1A–S1D). Treatment with 1,6-hexanediol (1,6-Hex), an inhibitor of phase separation, resulted in a reduced area of Venus-Snail1 condensates (Fig. S2A). To assess the fluidity of these condensates, we performed fluorescence recovery after photobleaching. The fluorescence intensity within Venus-Snail1 condensates dropped to ∼40% immediately after bleaching and subsequently recovered to nearly 80%. The quantitative analysis of the mobile fraction and half-recovery time (T_1_/_2_) of Venus-Snail1 condensates indicated their dynamic component exchange (Fig. S2B). Furthermore, immunofluorescence showed that the number of endogenous Snail1 condensates significantly increased after treatment with sorbitol in MHCC97H cells (Fig. S1E). These data support the conclusion that Snail1 protein has LLPS-like properties.

To identify the region mediating Snail1 LLPS-like properties, we constructed and expressed Snail1 truncations based on its domain architecture and predicted IDRs (Fig. 1A–C). Interestingly, none of the Snail1 truncations formed condensates. Only the deletion mutant lacking the IDR (ΔIDR) formed large, irregular aggregates that were distinct from the smaller, spherical condensates of wild-type Snail1 (Fig. 1D). Treatment with 1,6-Hex did not reduce ΔIDR aggregates (Fig. 1E). The fluorescence of ΔIDR aggregates did not recover after photobleaching (Fig. 1F). Together, these observations indicate that the ΔIDR mutant forms immobile, insoluble aggregates. We previously reported that protein dimerization or oligomerization may also mediate phase separation.^3^ To explore whether Snail1 protein can bind to itself, we performed GST pull-down assays in HEK293T cells co-transfected with GST-Snail1 and Venus-Snail1. Venus-Snail1, but not Venus alone, was detected in GST-Snail1, but not GST, protein complexes (Fig. S3A). To further investigate which domains mediate the binding of Snail1 to itself, we also performed GST pull-down assays in HEK293T cells co-transfected with GST-Snail1 and Venus-Snail1 truncations. The results showed that the ZFD region mediates the binding of Snail1 to itself (Fig. S3B and S3C). These data indicate that both the IDR and ZFD regions are involved in Snail1 LLPS-like properties.

**Figure 1:**
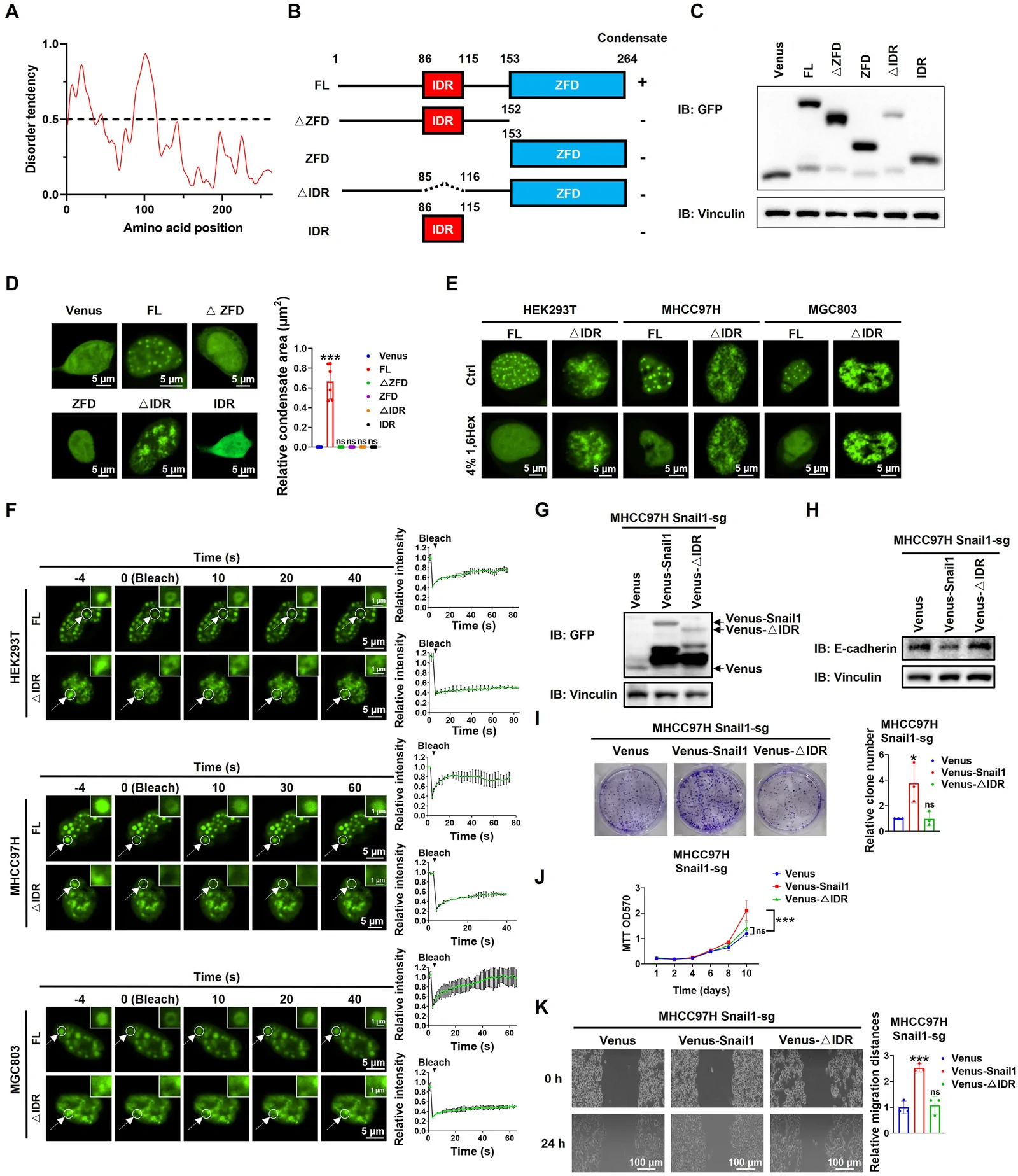
Phase separation of Snail1 modulates transcription and cancer progression. **(A)** The disorder tendency of human Snail1 protein predicted by IUPred3. **(B)** The diagrams of full-length and truncated Snail1. IDR, intrinsic disorder region; ZFD, Zinc finger domain. **(C)** The protein expression in the indicated cells. **(D)** HEK293T cells expressing Venus, Venus-Snail1 (FL), Venus-ΔZFD (ΔZFD), Venus-ΔIDR (ΔIDR), and Venus-IDR (IDR) were imaged and quantified (*n* = 6, one-way ANOVA). **(E)** HEK293T, MHCC97H, and MGC803 cells expressing Venus-Snail1 (FL) and Venus-ΔIDR (ΔIDR) were viewed after treated with 4% 1,6-hexanediol for 1 min. **(F)** Venus-Snail1 condensates and Venus-ΔIDR aggregates before and after fluorescence bleaching. **(G, H)** The protein expression in the indicated cells. **(I)** Colony formation images and quantitative results of the indicated cell (*n* = 3, one-way ANOVA). **(J)** Growth curves of the indicated cells (*n* = 3, one-way ANOVA). **(K)** Wound healing images and quantitative results of the indicated cells (*n* = 3, one-way ANOVA). ns, not significant. *P* > 0.05, ∗*P* ≤ 0.05, and ∗∗∗*P* ≤ 0.001.

To assess whether ΔIDR mutant differ from WT Snail1 in nuclear localization and protein stability, we stained nucleus with DAPI in HEK293T cells transfected with Venus-Snail1 or ΔIDR. The results showed that both Venus-Snail1 and ΔIDR localized in the nucleus, suggesting deletion of the IDR does not alter Snail1 nuclear localization (Fig. S4A). We then compared the half-life of Snail1 and ΔIDR in HEK293T cells transfected with Flag-Snail1 or ΔIDR. After treatment with the protein synthesis inhibitor cycloheximide (CHX) at the indicated time points, the half-life of Flag-Snail1 was shorter than that of ΔIDR, indicating deletion of the IDR increases Snail protein stability (Fig. S4B). Next, we co-expressed and examined the co-localization of ECFP-Snail1 and Venus-CDK7 (a core transcriptional component) in HEK293T cells. ECFP-Snail1 condensates, but not ΔIDR aggregates, were co-localized with Venus-CDK7 (Fig. S4C). Next, we compared the transcriptional activity of Snail1 and ΔIDR by co-transfecting them with the pEZX-FR01 vector, which contains Snail1-binding E-box motifs (CAGGTG), in HEK293T cells (Fig. S4D and S4E). The results of luciferase assays revealed that IDR deletion impaired the transcriptional activity of Snail1 (Fig. S4F). To further investigate the effect of Snail1 phase separation on cancer, we used the CRISPR/Cas9 system and successfully generated Snail1-knockout MHCC97H cells with deletion of 155bp in the genome (Fig. S5A and S5B). Snail1 knockout increased E-cadherin expression (Fig. S5C). A colony formation assay demonstrated that Snail1 knockout inhibited the colony formation of MHCC97H cells (Fig. S5D). MTT assays indicated that Snail1 knockout suppressed the growth of MHCC97H cells (Fig. S5E). Wound healing assays revealed that Snail1 knockout blocked the migration of MHCC97H cells (Fig. S5F). Then, Venus, Venus-Snail1, and Venus-ΔIDR were re-expressed in Snail1-knockout MHCC97H cells (Fig. 1G). Re-expression of Snail1, but not ΔIDR, decreased E-cadherin expression and enhanced colony formation, cell growth, and migration in Snail1-knockout MHCC97H cells (Fig. 1H–K). These findings link phase separation of Snail1, mediated by its IDR, to its transcriptional and oncogenic functions.

Collectively, our findings establish Snail1 as a protein capable of undergoing phase separation. This process, which requires both its IDR and ZFD regions, is essential for its transcriptional activity and its role in promoting oncogenic phenotypes such as enhanced proliferation, migration, and colony formation. However, deletion of the IDR may affect multiple aspects of Snail1 biology beyond condensate formation, including protein–protein interactions and chromatin association. Our study lacks mutants that selectively disrupt condensate dynamics without deleting the entire IDR. In addition, analysis of Snail1 occupancy at the CDH1 promoter under WT and ΔIDR rescue conditions will provide more support for transcriptional regulation by Snail1 condensates. These discoveries underscore the critical role of biomolecular condensation in Snail1-mediated gene transcription and cancer progression, suggesting novel therapeutic opportunities for targeting Snail1 and phase separation in cancer.

## CRediT authorship contribution statement

**Yi-Ping Huang:** Writing – original draft, Project administration, Methodology, Investigation. **Peng-Wei Zhang:** Writing – original draft, Project administration, Methodology, Investigation. **Ke He:** Writing – review & editing, Resources, Funding acquisition, Conceptualization. **Zhong-Bao Shao:** Validation, Methodology. **Wei-Jing Liu:** Investigation, Data curation. **You-Qing Zhu:** Validation, Resources. **Shi-Jie Zeng:** Methodology, Data curation. **Zhi Shi:** Writing – review & editing, Supervision, Funding acquisition, Conceptualization.

## Funding

This work was supported by the 10.13039/501100001809National Science Foundation of China (No. 82272996), 10.13039/501100010256Guangzhou Municipal Science and Technology Project, China (Nos. 202102070001, 202206010081, and 2024A03J0133), the 10.13039/501100002338Fundamental Research Funds for the Central Universities, China (No. 21625105), and the Support Project Fund of Guangdong Second Provincial General Hospital, China (No. TJGC-2023013).

## Conflict of interests

Zhi Shi is an Associate Editor of *Genes & Diseases* and was not involved in the editorial review or the decision to publish this article. The remaining authors declare that there are no competing interests.
