# Supplementary material for: Phase separation of Snail1 modulates transcription and cancer progression
Source: Genes Dis. 2026 Mar 17;14(3):102147. doi: 10.1016/j.gendis.2026.102147 (PMC13734866; doi:10.1016/j.gendis.2026.102147)
Supplement: Supplementary file 1 [file mmc1.docx]

**Materials and methods**

**Cell lines**

Human embryonic kidney cells HEK293T, human hepatocellular carcinoma cells MHCC97H, and human gastric cancer cells MGC803 (from the Cell Bank of the Chinese Academy of Sciences) were grown in DMEM supplemented with 10% fetal bovine serum (SA301.02.V, CellMax), 100 µg/mL streptomycin, and 100 U/mL penicillin at 37 ºC with 5% CO_2_ in a humidified incubator.

**Reagents**

PEG8000 (A660434), sorbitol (A555023) and anti-GFP antibody (D110008) were purchased from Sangon Biotech (Shanghai, China). 1,6-hexanediol (1,6 Hex, Q106706), G418 (H756005) and methyl thiazolyl tetrazolium (MTT, Q108115 ) were purchased from D&B Biotechnology (Shanghai, China). Polyetherimide (PEI, 23966-1) was purchased from Polysciences (Warrington, PA, USA). Cycloheximide (CHX, 02194527) was purchased from MP Biomedicals (Santa Ana, CA, USA). Anti-Snail1 antibody (13099-1-AP) was purchased from Proteintech (Wuhan, China). Anti-GST antibody (CSB-MA000031M0m) was purchased from Cusabio (Wuhan, China). Anti-Vinculin antibody (BM1611) was purchased from BOSTER (Wuhan, China). Anti-α-tubulin antibody (sc-5286) was purchased from Santa Cruz Biotechnology (Shanghai, China). Anti-β-tubulin antibody (30302ES60) and anti-Flag antibody (30505ES) were purchased from Yeasen Biotechnoloy (Shanghai, China). Anti-E-cadherin antibody (3195) was purchased from Cell Signaling Technology (Boston, MA, USA). Anti-HSP90 antibody (610419) was purchased from Abcam (Cambridge, UK).

**Plasmids**

All Snail1 and CDK7 constructs were generated using Gateway cloning technology with pDEST27-GST, pCDH-Neo-Venus/Dest, pDEST-3×Flag, or pDEST-ECFP as destination vectors.Based on full-length human Snail1 (264 aa, Gene ID: 6615), we produced the following variants: Zinc finger domain (ZFD, 153–264), ΔZFD (deletion of aa 153–264), IDR (85–116), and ΔIDR (deletion of aa 85–116). The primers used for these constructions were as follows: for the ΔZFD, forward 5'-GCTCGAAAGTGAAACCCAGCTTTCTTGTACAAAG-3' and reverse 5'-GCTGGGTTTCACTTTCGAGCCTGGAGATCC-3'; for the ZFD, forward 5'-GCAGGCTTAGCCTTCAACTGCAAATACTGCAAC-3' and reverse 5'-CAGTTGAAGGCTAAGCCTGCTTTTTTGTACAAAC-3'; for the ΔIDR, forward 5'-CCAGGGTGACTTCAGTCTCTTCCTTGGAG-3' and reverse 5'-GACTGAAGTCACCCTGGGACTCTCCTG-3'; and for the IDR, forward 5'-GGGGACAAGTTTGTACAAAAAAGCAGGCTTAATGgcagagctgacctccctg-3' and reverse 5'-GGGGACCACTTTGTACAAGAAAGCTGGGTTTTAagaggagaaggacgaaggagccg-3'.

**Fluorescence recovery after photobleaching (FRAP)**

FRAP assays were performed using a Zeiss LSM900 confocal microscope (Oberkochen, Germany). Selected condensates were photobleached using a 488 nm laser at 100% laser power. Imaging is performed at specified intervals after bleaching. The mobile fraction was calculated as (I_∞_ – I_0_) / (I_pre_ – I_0_). I_pre_, I_0_, and I_∞_ indicate the fluorescence intensity before bleaching, immediately after bleaching, and at the plateau, respectively. T₁/₂ is the time for intensity to recover to half of its final value from the post-bleach minimum.

**Immunofluorescence assay**

After removal of the medium, cells on the coverslides were fixed with 4% paraformaldehyde for 20 minutes and then washed three times with PBS for 5 minutes each. Cells were permeabilized with 0.5% Triton X-100 and then blocked for 1 hour using 2% BSA at room temperature. Subsequently, cells were immersed in their primary antibody overnight at 4 ºC and washed three times with PBS. After the addition of fluorescent secondary antibodies, cells were incubated in the dark for 1 hour, followed by three washes with PBS. After 15-minute incubation with DAPI, the coverslides were washed three times with PBS, sealed with anti-fluorescence quencher, and the imaged were captured with a Zeiss LSM900 confocal microscope (Oberkochen, Germany).

**Knockout cell lines and stable overexpressing cell lines**

Knockout cell lines were generated using the lentiviral CRISPR/Cas9 system. The sgRNA targeting Snail1 (Snail1 sgRNA-F: 5'-CACCGcttccagcagccctacagc-3'; Snail1 sgRNA-R: 5'-AAACggtcgtagggctgctggaagC-3') was designed, synthesized, and cloned into the LentiCRISPR V2 vector (#52961, Addgene). The vector was then co-transfected with pMD2.G and psPAX2 into HEK293T cells to package lentivirus. MHCC97H cells were infected with lentiviruses concentrated by ultrafiltration tubes (FTT400500, Jet Biofil). Monoclonal cells were selected with puromycin (P113126, Aladdin), and the knockout efficiency was verified by Western blot. The genomic DNA of selected clones was extracted, amplified, and sequenced by the following primers: forward 5'-AGTACTTAAGGGAGTTGGCGG-3' and reverse 5' -CTCGGCCTCCAAGGAAGAG-3'. For stable overexpression,  pCDH-Neo-Venus, pCDH-Neo-Venus-Snail1, and pCDH-Neo-Venus-ΔIDR vectors were co-transfected with pMD2.G and psPAX2 into HEK293T cells for lentivirus packaging. The Snail1-knockout cells were infected with these lentiviruses, selected with G418, and overexpression was verified by Western blot.

**Western blot**

Cell lysates containing equal amounts of protein were separated by 8–12% SDS-PAGE and transferred onto polyvinylidene difluoride membranes (IPVH00010, Merck Millipore). The membranes were blocked with 5% skimmed milk for 1 hour at room temperature and then incubated with the appropriate primary antibody overnight at 4 °C. After washing, the membranes were incubated for 1 hour at room temperature with horseradish peroxidase-conjugated secondary antibodies against rabbit or mouse (Beyotime, Shanghai). After developing with  enhanced chemiluminescence (JXE0011, Jingxin), signals were detected using a ChemiDoc XRS chemiluminescence imaging system (Analytik Jena, Jena, Germany).

**GST pull-down assay**

HEK293T cells were co-transfected with the indicated vectors using PEI. After 48 hours of culture, cells were collected and lysed on ice. Following centrifugation, the supernatant was incubated with 40 µL GST-Tag Purification Resin (P2251, Beyotime) overnight at 4°C with rotation. The resin was then washed extensively with ice-cold lysis buffer, resuspended in 2× SDS loading buffer, boiled at 100°C for 10 minutes, and the eluted proteins were analyzed by Western blot.

**MTT assay**

Cells were seeded at a specified density in 96-well plates (TCP010096, Jet Biofil). At the indicated time points, MTT solution was added to a final concentration of 0.5 mg/mL. After 4 hours of incubation, the supernatant was carefully removed, and the formazan crystals were dissolved in DMSO (D10042, Psaitong). The absorbance at 570 nm was measured.

**Colony formation assay**

Cells were seeded at a low density in 6-well plates (TCP010006, Jet Biofil) and cultured for 15 days. They were then fixed with 4% paraformaldehyde for 15 minutes and stained with 0.5% crystal violet for 30 minutes. The colonies were photographed and counted under the microscope.

**Wound healing assay**

Cells were grown to 90% confluence in 6-well plates (TCP010006, Jet Biofil) and a scratch wound was created with a 200 µL pipette tip. After washing with PBS to remove debris, cells were maintained in serum-free medium. Images of the scratch were captured at 0 and 24 hours, and cell migration was assessed by measuring the relative wound width using ImageJ software.

**Luciferase reporter assay**

HEK293T cells were co-transfected with the pEZX-FR01 luciferase reporter vector (GeneCopoeia, Guangzhou, China) and the indicated vectors using PEI. After 48 hours, cells were collected, washed twice with PBS, and lysed using passive lysis buffer (RG126M, Beyotime) for 10 minutes. For the firefly luciferase activity assay, a reaction solution (containing 30 µg/mL luciferin, 100 µM ATP, 100 µM Mg²⁺, 100 µM Coenzyme A) was mixed with the lysate at a 10:1 ratio. For Renilla luciferase activity (internal control), 1 µM coelenterazine was added at a 1:10 ratio. Luminescence was measured immediately using a luminometer.

**Statistical Analysis**

Data are presented as mean ± standard deviation. Statistical analyses were performed using GraphPad Prism 8. The unpaired Student's t-test and one-way ANOVA were used to compare two groups and multiple groups, respectively. A P value of < 0.05 was considered statistically significant, denoted as * *P*< 0.05, ** *P*< 0.01, and *** *P*< 0.001.

**Fig. S1** Snail1 has LLPS-like properties. **(A, B)** HEK293T, MHCC97H, and MGC803 cells expressing Venus and Venus- Snail1 were imaged and the condensate area was quantified after treated with 0.2 M sorbitol for 5 minutes and 10% PEG8000 for 3 minutes (n=6, unpaired t-test). **(C, D)** Time-lapse images showing fusion events of Venus-Snail1 condensates. **(E)**  Immunofluorescence staining of endogenous Snail1 in MHCC97H cells treatment with 0.8 M sorbitol for 20 minutes. Nuclei were stained with DAPI (blue). Quantification of Snail1-positive condensates per cell is shown on the right (n = 6, unpaired t-test). * *P* ≤ 0.05, ** *P* ≤ 0.01, and *** *P* ≤ 0.001 vs. corresponding group.
**Fig. S2** Snail1 forms dynamic condensates. **(A)** HEK293T, MHCC97H, and MGC803 cells expressing Venus-Snail1 were imaged at the indicated time points after treatment with 4% 1,6-Hex. The relative condensate area was quantified over time (n=6, group t-test). **(B)** Representative FRAP images of a Venus-Snail1 condensate (pre-bleach, post-bleach, and recovery). The graph shows the quantitative fluorescence recovery curve. The mobile fraction and half-recovery time (T₁/₂) were calculated as described in the Methods. * *P* ≤ 0.05, ** *P* ≤ 0.01, and *** *P* ≤ 0.001 vs. corresponding group.

**Fig. S3** The ZFD mediates Snail1 self-interaction. (A, B) Western blot analysis of inputs and GST pull-down products from HEK293T cells co-expressing the indicated GST- and Venus-tagged proteins. (C) Schematic diagram of full-length and truncated Snail1 constructs used in this study**.**

**Fig. S4** The IDR is required for Snail1 transcriptional activity. (A) Subcellular localization of Venus-Snail1 and Venus-ΔIDR in HEK293T cells. Nuclei were stained with DAPI (blue). (B) Western blot analysis of Flag-Snail1 and Flag-ΔIDR protein levels in HEK293T cells treated with 50 µg/mL cycloheximide (CHX) for the indicated times. β-tubulin served as a loading control. **(C)** Co-localization analysis of Venus-CDK7 and ECFP-Snail1 or ECFP-ΔIDR in HEK293T cells. **(D)** Schematic of the pEZX-FR01 luciferase reporter vector containing Snail1-binding E-box motifs. **(E)** Western blot showing expression levels of the indicated proteins in HEK293T cells used for the luciferase assay. **(F)** Relative luciferase activity of the pEZX-FR01 reporter in HEK293T cells expressing the indicated proteins (n=5, one-way ANOVA). ns *P*> 0.05, and *** *P* ≤ 0.001 vs. corresponding group.

**Fig. S5** Snail1 knockout suppresses oncogenic phenotypes in MHCC97H cells. (A) Western blot analysis of Snail1 expression in control and Snail1-knockout (KO) MHCC97H cells. α-Tubulin served as a loading control. (B) Representative genomic DNA sequencing chromatograms aligning the target region in control and Snail1-KO clones. (C) Western blot analysis of E-cadherin expression in control and Snail1-knockout (KO) MHCC97H cells. Vinculin served as a loading control. (D) Representative images (left) and quantification (right) of colony formation assays (n=3, unpaired t-test). (E) Cell growth curves determined by MTT assay (n=3, unpaired t-test at day 9). (F) Representative images (left) and quantification (right) of wound healing assays at 0 and 24 hours (n=3, unpaired t-test). * *P* ≤ 0.05, and ** *P* ≤ 0.01 vs. corresponding group.
